# Relationship Between Social Anxiety and Internet Addiction in Chinese College Students Controlling for the Effects of Physical Exercise, Demographic, and Academic Variables

**DOI:** 10.3389/fpsyg.2021.698748

**Published:** 2021-08-05

**Authors:** Songdong Ye, Huiying Cheng, Zongpeng Zhai, Hongyou Liu

**Affiliations:** School of Physical Education & Sports Science, South China Normal University, Guangzhou, China

**Keywords:** physical activity, gender difference, academic career, family status, discipline and specialty, university students

## Abstract

This study aims to identify the relationship between social anxiety (SA) and internet addiction (IA) in a group of Chinese college students by controlling for the effects of physical exercise (PE), demographic, and academic variables. A sample of 4,677 students from five major regions of China participated in this survey. The findings revealed that: (1) SA had a direct effect on IA; (2) regular and active participation in physical exercise can relieve SA and IA effectively; (3) the level of SA and IA is strongly linked to sex; (4) the levels of SA and IA are different among students of different majors; (5) students in the middle phase of their academic career are more likely to have IA than those in the starting phase. The study is significant because few existing studies discuss the role of PE on SA and IA. Additionally, the study found that college students with more PE would have a lower level of SA and a lower probability of IA.

## Introduction

Internet addiction (IA) caused by the overuse of computers and smartphones has become a major public health problem worldwide, especially for the youth (Cash et al., 2012; Festl et al., 2013; Eliahu, 2014; OECD, 2017). The global prevalence of IA ranges from 1.6 to 18% (Shaw and Black, 2008), and can vary by age, sex, and race, and is more common among college students (Pujazon-Zazik and Park, 2010). In 2018, approximately 77% of U.S. residents owned smartphones (Pew Research Center, 2018). Moreover, Smith and Page (2015) reported that 46% of U.S. residents expressed that they could not live without their smartphones. This phenomenon has also been observed in other Western countries (OECD, 2017). According to the 46th statistical report of the China Internet Network Information Center (CINIC) in 2020, as of June 2020, the number of Chinese Internet users reached 940 million. Further, mobile Internet users reached 932 million, while the proportion of Internet users using mobile phones reached 99.2% (CINIC, 2020). Young people (18–25 years old) have clearly become the largest user group among mobile Internet users, accounting for more than a third of the total number, most of whom are college students (Yang et al., 2019). Currently, the problem of IA of college students caused by the excessive use of smartphones is a hot research topic (Amez and Baert, 2020).

Several studies have investigated the negative effects of IA (including smartphone use) on academic performance, sleep quality, depression, anxiety, and physical fitness. It has been identified that there is a negative correlation between the daily time spent on smartphones and academic performance; specifically, using a smartphone for 100 min on an average per day corresponded to a 6.3 point reduction in the position of a student in the ranking in school in Brazil (Felisoni and Godoi, 2018). Moreover, the relationship between smartphone addiction scale scores and depression levels, anxiety levels, and some sleep quality scores indicated that smartphone overuse might lead to depression or anxiety, which might, in turn, cause sleep problems (Demirci et al., 2015). A survey of 119 Japanese university students showed that smartphone addiction and poor sleep quality were correlated with the state of depression among students in Japan (Ezoe et al., 2019). To a large extent, IA reduces the time of participation in physical exercise by college students and increases sedentary behavior, resulting in insufficient physical activity, and eventually affects their fitness and health (Lepp et al., 2013; Kim et al., 2015; Barkley and Lepp, 2016).

In addition to IA, social anxiety (SA) is considered another common and debilitating psychiatric disorder in college students (Russell and Shaw, 2009). According to the American Psychiatric Association (2013) SA is “characterized by a marked and persistent fear of one or more social situations (e.g., talking to a stranger or peer, going to a party) or performance activities (e.g., giving a speech) in which the person is exposed to unfamiliar people, or where they may face possible scrutiny by others.” SA has an early onset age with a high comorbidity with other psychiatric disorders and subsequent evident impairment (Kessler et al., 2009). The prevalence of SA among college students was high. Studies have shown that 10% of college students in the United Kingdom suffer from SA (Russell and Shaw, 2009). Further, 30% of newcomers suffered from SA in Australia (Wilson, 2005), while the percentage of SA among students in medical specialties in Iraq, Kingdom of Saudi Arabia, and India was 28.3, 29.8, and 30.5%, respectively (Ahmad et al., 2017; Hakami et al., 2017; Jarallah et al., 2017; Dsouza et al., 2019; Reta et al., 2020). SA has a significant negative impact on interpersonal relationships, work, and academic performance, such as increasing the risk of dropping out of school, reducing work efficiency, and affecting the quality of life (Aderka et al., 2012; Jacobson et al., 2020). If left untreated, SA can often lead to mental health problems, including severe depression, substance abuse, and cardiovascular disease (Dryman et al., 2016; Leichsenring and Leweke, 2017).

Physical exercise may positively treat both IA and SA (Liu et al., 2019). For example, Jazaieri et al. (2012) suggested that aerobic exercise is associated with reductions in SA and depression and increases in subjective wellbeing. Pan (2020) found that exercise intervention mixed with psychological means decreased IA in teenagers. Lin et al. (2020) demonstrated that physical exercise could mediate the relationship between IA and psychological and physical symptoms. Indeed, the impact path from PE to IA or SA was through exercise time, intensity, or frequency (Chen et al., 2020). For example, Liu et al. (2019) found that the longer the duration of physical exercise, the greater the therapeutic effect. More specifically, Paolucci et al. (2018) found that moderate-intensity exercise may be an optimal intensity of exercise for promoting mental health by decreasing tumor necrosis factor α (TNF-α), which is critical for informing the use of exercise as a medicine for mental health. However, this exercise of moderate-intensity has to be limited to a certain duration (45 min) and frequency (3–5 times per week) (Chekroud et al., 2018).

Some limitations should be acknowledged while exploring IA or SA. First, the most existing studies focus more on the harm and influence of IA or SA, respectively, and few studies have focused on the impact of PE on IA and SA. For example, Yang et al. (2019) analyzed the relationship between PE, mobile phone dependence (MPD), and self-control. However, the sample volume of that study was limited (650 college students in a specific area in China), which reduced its representativeness. Furthermore, Zou et al. (2018) explored the relationship between PE and SA in subjects consisting of only children. Similarly, Ren and Li (2020) found that PE has a significant impact on the SA of left-behind children in rural Chinese areas. Moreover, some previously mentioned researchers from the United Kingdom, Austria, Iraq, Saudi Arabia, India, and Ethiopia conducted studies in their own countries (Wilson, 2005; Russell and Shaw, 2009; Ahmad et al., 2017; Hakami et al., 2017; Jarallah et al., 2017; Dsouza et al., 2019; Reta et al., 2020). However, few studies have examined the relationship between PE, IA, and SA in a group of Chinese college students. Additionally, demographic and academic variables of students should seriously be considered because the degree of IA and SA of individuals is not similar when accounting for variables such as sex, major, grade, and age. Therefore, further research is still needed to better understand the relationship between the PE, IA, and SA of students in different situations.

Accordingly, this study aims to utilize the generalized mixed linear model to examine the relationship between IA and SA, taking into account the variables of PE, sex, major, grade, and age among Chinese college students. The results of this study can help reduce the levels of IA and SA in Chinese college students with different backgrounds through PE approaches. Based on the reviewed literature, this study hypothesized that: (1) A positive relationship would exist between IA and SA. (2) PE would be negatively associated with IA and SA.

## Materials and Methods

### Subjects and Procedures

A self-reported standard scale was used for the cross-sectional survey in this study. Further, the survey was conducted from April 9th to 23rd, 2020. A stratified cluster sampling method was adopted to acquire subjects. According to the existing criterion, the territory of China can be divided into five regions (i.e., east, west, south, north, and central). Twenty major cities in these five regions were selected, and each region included four cities. Sequentially, 42 universities were chosen. Each class of these universities has been viewed as a cluster. Further, the sample has been randomly selected from all the classes of these universities. All currently selected students who were able to complete the questionnaire independently were included in our sample, composed of 4,677 participants, including undergraduates and postgraduates. All 4,677 college students participated in the survey, which was completed anonymously and confidentially online, and all questionnaires were collected voluntarily. After further examination, 30 questionnaires were found to be invalid due to missing data or incorrect or incomplete answers and hence were deleted from the sample. The age, sex, and major distribution of the final sample are shown in Table 1. The Ethics Committee of the School of Physical Education and Sports Science of South China Normal University approved this study.

**TABLE 1:** Descriptive data of the predictor and dependent variables.

Numeric (mean ± SD)								
PE	19.0 ± 21.4							
SA	42.3 ± 8.3							
Age	20.0 ± 2.2							

**Nominal [frequency (%)]**								

IA	NIA	IA						
	3,439 (74.00)	1,208 (26.00)						
Gender	Male	Female						
	1,814 (39.04)	2,833 (60.96)						
Single child	SC	NSC						
	1,232 (26.51)	3,415 (73.49)						
Major	SS	NS	Arts	Sports				
	1,631 (35.10)	1,945 (41.85)	281 (6.05)	790 (17.00)				
Grade	UG1	UG2	UG3	UG4	PG1	PG2	PG3	DR
	2664 (57.33)	1375 (29.59)	179 (3.85)	96 (2.07)	196 (4.22)	83 (1.79)	29 (0.62)	25 (0.54)

**PE, physical exercise; SA, social anxiety; IA, internet addiction; NIA, no internet addiction; SC, single child; NSC, non-single child; SS, social science; NS, natural science; UG1-UG4, 1st–4th year of undergraduate; PG1-PG3, 1st–3rd year of postgraduate; DR, doctorate.**

### Measures

#### Physical Activity Rating Scale-3

Physical exercise was measured using the Physical Activity Rating Scale-3 (PARS-3) (Liang and Liu, 1994). Its validity and reliability have been demonstrated in many studies in China (Yang et al., 2019). The PARS-3 is a three-item, self-reported scale that contains exercise intensity, duration, and frequency. The internal consistency reliability (Cronbach’s alpha) of PARS-3 was 0.76. The PARS-3 has excellent test-retest reliability (*r* = 0.82) (Sun and Yang, 2015).

#### Internet Addiction Diagnostic Questionnaire

Internet addiction was assessed using the Internet Addiction Diagnostic Questionnaire (IADQ) (Young, 1996). We chose the IADQ because it is a short questionnaire widely used in research examining the prevalence of IA across cultures, especially in the extant literature examining the prevalence and correlations of IA/Problematic Internet Use among youth and young adults (Li et al., 2015). The IADQ contains eight questions. Participants who answered “yes” to five or more of the criteria were diagnosed with IA. According to prior studies, the split-half reliability of IADQ reached 0.729 (Johansson and Götestam, 2004). Simultaneously, the internal consistency reliability (Cronbach’s alpha) of the IADQ was 0.72 (Dowling and Quirk, 2009), which demonstrated that IADQ could display good reliability, consistency, and unidimensionality (Johansson and Götestam, 2004).

#### Interaction Anxiousness Scale

In testing the degree of SA, this study uses the Interaction Anxiousness Scale (IAS) (Leary, 1983), which is composed of 15 self-reported test questions. IAS is a five-level self-rating scale, with scores from one to five, from “not at all consistent” to “extremely consistent,” in which the 3rd, 6th, 10th, and 15th questions are reverse scored. The IAS has a high internal consistency coefficient (α = 0.89) and test-retest reliability (*r* = 0.80) (Leary, 1983).

### Statistical Analysis

Generalized mixed linear modeling was performed using Proc Glimmix in the University Edition of Statistical Analysis System (version SAS Studio 3.6). Two separate regressions were run in the model. First, Poisson regression was achieved by taking SA as the dependent variable and PE and IA as predictor variables. Second, a binomial logistic regression was employed by taking IA as the dependent variable and PE and SA as predictor variables. Fixed effects of sex, age, single child (SC), major, and grade were added to both regression models.

Social anxiety is the summed value of all items in the IAS, with a possible range of 15–75. PE was calculated from the three items of the PARS-3 using the following equation: exercise intensity × (exercise duration-1) × exercise frequency, with a possible range of 0–100 (Yang et al., 2019). IA was included as a nominal variable with two levels (Internet addiction, IA > 4 and no Internet addiction (NIA), IA ≤ 4). Sex and SC were included as nominal variables with two levels as well (male and female, single child, and non-single child (NSC), respectively). Major was added as a four-level nominal variable (social science, natural science, arts, and sports). The grade was added as a seven-level nominal variable (1st year of undergraduate (UG1), 2nd year of undergraduate (UG2), 3rd year of undergraduate (UG3), 4th year of undergraduate (UG4), 1st year of postgraduate (PG1), 2nd year of postgraduate (PG2), 3rd year of postgraduate (PG3), and doctorate (DR). Age was included as a numeric variable using raw values.

In the Poisson regression model, the differences in the mean SA for all groups of nominal variables were compared. For the effects of PE and age (numeric variables), the difference in the mean SA of a typically low value (one SD below the mean) and a typically high value (1 SD above the mean) was estimated (Hopkins et al., 2009). In the binomial logistic regression model, the differences in the probability of IA for all groups of nominal variables were compared. The change in the probability of IA from a typically low value (1 SD below the mean) to a typically high value (1 SD above the mean) of PE, SA, and age was estimated. The difference in the mean and the probability, and their 95% compatibility intervals, are presented as percentages (%). Only the differences with a significance level of less than 0.05 are discussed in this study.

## Results

**FIGURE 1:**
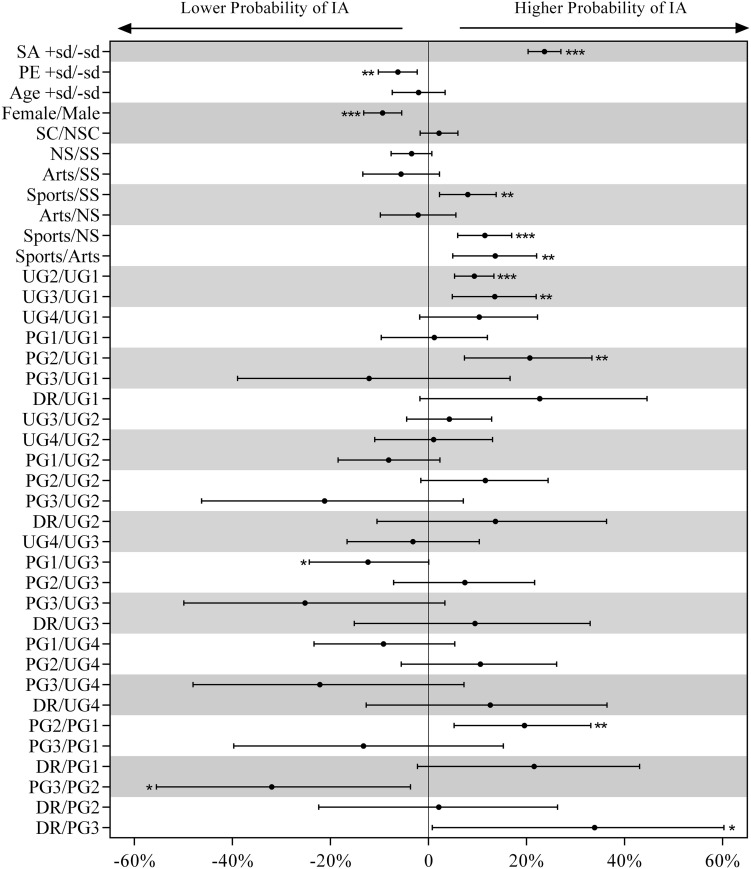
Effects of IA, PE, age, gender, single child, major, and grade on the SA estimated from the Poisson regression. Dots are the effects in percent units, and error bars are their 95% compatibility intervals. Asterisks indicate the significance level as follows: *p < 0.05, **p < 0.01, ***p < 0.001. PE, physical exercise; SA, social anxiety; IA, internet addiction; NIA, no internet addiction; SC, single child; NSC, non-single child; SS, social science; NS, natural science; UG1-UG4, 1st–4th year of undergraduate; PG1-PG3, 1st–3rd year of postgraduate; DR, doctorate.

**FIGURE 2:**
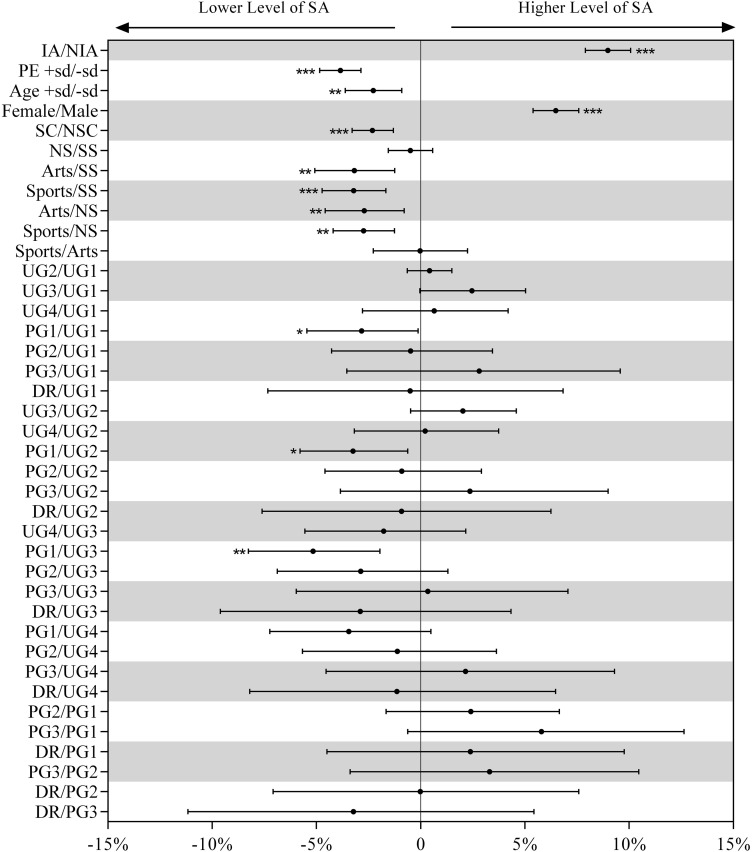
Effects of SA, PE, age, gender, single child, major, and grade on the IA estimated from the binomial logistic regression. Dots are the estimated difference in the probability of IA, and error bars are their 95% compatibility intervals. Asterisks indicate the significance level as follows: *p < 0.05, **p < 0.01, ***p < 0.001. IA, internet addiction; PE, physical exercise; SA, social anxiety; SC, single child; NSC, non-single child; SS, social science; NS, natural science; UG1-UG4, 1st–4th year of undergraduate; PG1-PG3, 1st–3rd year of postgraduate; DR, doctorate.

Descriptive statistics of the dependent and independent variables are shown in Table 1. Moreover, the relationship between SA, IA, and the PE and demographic and academic variables estimated from the generalized mixed linear modeling is presented in Figures 1, 2.

Results from the Poisson regression showed that the SA level of the students with IA was higher (+ 8.98%; 95%CI: ± 1.09%, *p* < 0.0001) than the students with NIA. The level of SA of the students with high (1 SD above the mean) PE values was lower (−3.85%; ± 0.99%, *p* < 0.0001) than the students with low (1 SD below the mean) PE values. Students with an age of 1 SD above the mean age had a lower SA level of 2.27% (± 1.35%, *p* = 0.0011) than those aged below the mean. Female students had a higher SA level (+ 6.48%; ± 1.10%, *p* < 0.0001) than male students. Single child students had a lower level (−2.31%; ± 0.99%, *p* < 0.0001) of SA than NSC. The SA levels of students majoring in arts were lower than those majoring in social sciences (−3.18%; ± 1.92%, *p* = 0.0014) and natural sciences (−2.70%; ± 1.90%, *p* = 0.0057). Meanwhile, the SA levels of students majoring in sports were also lower than those majoring in social sciences (−3.21%; ± 1.53%, *p* < 0.0001) and natural sciences (−2.74%; ± 1.48%, *p* = 0.0003). The level of SA of students of PG1 were lower than students of UG1 (−2.83%; ± 2.67%, *p* = 0.0411), UG2 (−3.24%; ± 2.58%, *p* = 0.0156), and UG3 (−5.16%; ± 3.16%, *p* = 0.0018).

Results from the binomial logistic regression showed that the college students, with a SA level at 1 SD above the mean level, had a 23.67% (± 3.34%, *p* < 0.0001) higher probability of IA than those with a SA level under the mean. Students with a PE level above the mean would have a 6.28% (± 3.96%, *p* = 0.0019) lower probability of IA than those with a PE level below the mean. Female students had a lower probability of IA than male students (−9.38%; ± 3.89%, *p* < 0.0001). Students majoring in sports had a higher probability of IA than students majoring in social sciences (+ 8.02%; ± 5.78%, *p* = 0.0068), natural sciences (+ 11.48%; ± 5.49%, *p* ≤ 0.0001), and arts (+ 13.58%; ± 8.55%, *p* = 0.0022). Students in UG2 (+ 9.32%; ± 4.00%, *p* < 0.0001), UG3 (+ 13.50%; ± 8.56%, *p* = 0.0023), and PG2 (+ 20.69%; ± 12.99%, *p* = 0.0026) were more likely to have IA than students in UG1. PG1 students had a lower probability of IA than students in UG3 (−12.34%; ± 12.19%, *p* = 0.0507) and PG2 (−19.55%; ± 13.93%, *p* = 0.0077). PG3 students had a lower probability of developing IA than students in PG2 (−32.00%; ± 25.92%, I = 0.0273) and DR (−33.85%; ± 29.74%, *p* = 0.0451).

## Discussion

The current study identified the relationship between SA and IA among Chinese college students accounting for the effects of PE, sex, age, SC, major, and grade by utilizing the generalized mixed linear model.

In general, our results showed that the Chinese college students with IA had a significantly higher SA than the students without IA; correspondingly, the students with a higher level of SA had a greater possibility of IA. This result is supported by a previous study conducted by Ko et al. (2008). They recruited 216 college students (132 men, 84 women) and evaluated the association between IA and depressive disorder, social phobia, and adult attention deficit hyperactivity disorder (ADHD), examining sex differences. Their results indicated that IA of college students is associated with SA, and the level of SA is a predictor of IA (Ko et al., 2008). Therefore, individuals with SA symptoms are more likely to have IA, which increases the severity of SA (Dong et al., 2019).

Furthermore, our survey showed that PE is negatively correlated with SA and IA, which means that college students with more PE would have lower levels of SA and a lower probability of IA. This result is in line with the findings of Rodebaugh et al. (2007) and Jazaieri et al. (2012), who found that aerobic exercise and mindfulness-based stress reduction (MBSR) resulted in similar, significant improvements in measures of SA, depressive symptoms, and wellbeing at post-intervention and 3-month follow-up. Therefore, this study offers preliminary and partial support for the efficacy of aerobic exercise in the treatment of SA. Possibly, a supervised intervention would produce more robust effects (Asmundson et al., 2013). Furthermore, Lin et al. (2020), who carried out a study of 1,854 students from 11 middle and high schools in Shenzhen, Guangdong Province, China, found that sports can improve wellbeing of students and significantly improve SA symptoms and depression. Thus, PE alleviates the relationship between psychological and physical symptoms, indicating that PE can reduce the negative impact of IA on health. However, physical activity does not mean positive results unless under an appropriate exercise intensity, duration, and frequency, because in the long term, vigorous activity could increase psychological burden of people. This, in turn, aggravates their depressive disorder (Chekroud et al., 2018). Moreover, competition is a normal form of PE in college physical education curriculums, and it has a positive influence on the physical and psychological health of participants when properly organized (Strong et al., 2005; Appelqvist-Schmidlechner et al., 2018). Nevertheless, it is noted that organizers should not neglect the negative effects that competition may bring to participants because competition itself has a certain selectivity and exclusivity, such as sex and skill level (Gould and Carson, 2008; Ortega Vila et al., 2020). Hence, students suffering from SA or IA should be aware that PE must be conducted appropriately, based on their own physical condition and level of SA or IA.

The results of our model also quantified the effects of demographic and academic variables on SA and IA. Considering the effect of sex, we found that female students had higher SA levels than their male counterparts, which is in line with prior research (Mclean et al., 2011), showing that women had higher scores than men in both general and various other types of anxiety. Furthermore, according to Rana et al. (2013), most epidemiological and community surveys have found that women have a higher prevalence of SA. However, our results show that male students are more prone to IA than women. This may be because men indulge in more frequent and high-risk Internet behaviors—including addiction to online games and gambling and watching pornographic materials—while women indulge in fewer high-risk Internet behaviors (Tsitsika et al., 2009). Therefore, psychologists should pay more attention to the “spiritual life” of a male, creating an atmosphere with less competitiveness, especially in some universities with a higher density of women, such as a girl’s or regular college. On the contrary, in sports schools or universities that are “dominated” by men, managers or counselors should strengthen the management and supervision of online activities of students, helping them stay away from dangerous online activities or a long-term addiction to the virtual world.

Accounting for the family effects, we find that the SA level of single child is lower than that of the non-single child, which is consistent with the previous finding that SCs have the lowest rate of SA (15.6%) and the middle child had a higher prevalence rate (61.5%) (Hakami et al., 2017). Due to the family structure, the relationship between the children of non-single child families is more diverse, especially middle children who cannot get too much care from their parents, making them more prone to anxiety (Hakami et al., 2017). However, children from single-child families can receive sufficient care from their parents and have a strong sense of self-identity and a low degree of SA (Li, 2011). Therefore, with implementing the two-child policy in China, both the government and schools should pay more attention to the mental health of children from non-single child families and appeal strongly to parents to care for every child equally.

When considering the effects of majors of college students, our results showed that the SA level of students majoring in arts and sports is lower than that of students majoring in natural and social sciences, which is supported by prior research (Ermis and Imamoglu, 2019). This result is likely a consequence of students who majored in arts and sports often go out of the “classroom” to participate in different social environments at different times for competitions and communicate with athletes and people from different cultures, making them feel the confidence that arts or sports gives them (İmamoğlu et al., 2018). Meanwhile, by shaping appearance or posture through arts or sports activities such as dancing or playing basketball, PE has a positive influence on the SA of participants (Marquez and McAuley, 2001; Edwards et al., 2005). Interestingly, students majoring in sports are more likely to be addicted to the Internet than students majoring in other subjects, probably because they prefer to play video games in their leisure time and are easily addicted to them (Carver and Connor-Smith, 2010).

The effects of age and grade were investigated. We find that the SA of students of PG1 is lower than that of UG1, UG2, and UG3, which is partially consistent with the research of Reta et al. (2020), which showed that SA would increase with the increase in grade because senior students may be facing the pressures of the dissertation, employment, or further education, which may aggravate SA. However, it is, to some extent, contrary to the study of Rabie et al. (2019) and Desalegn et al. (2019), which indicated that SA would decrease with an increase in grade. These results could be due to university settings where “younger” students are forced to live far away from their parents for the first time and are exposed to new environmental stressors, including new social situations (Sadock et al., 2015; Djidonou et al., 2016). Moreover, living in a new environment, stress, and environmental factors play a role in interpersonal stressors. Thus, they can contribute to the development of SA and differences in background, appearance, language, social, and emotional development, all of which could affect whether or not a student fits in the university (Yen et al., 2010). Therefore, administrative departments such as admission offices should unite with student unions to create a comfortable atmosphere of enrollment. Further, they should give them advice and organize group activities (e.g., ice-breaking activity) during their first months, to help them adjust to their new college life as soon as possible. Meanwhile, parents should keep in touch with their children regularly, care for them, and prevent potential mental issues that could be caused by an unfamiliar interpersonal environment.

Additionally, this study shows that students in the middle of the academic period (UG2, UG3, and PG2) are more likely to develop IA than students at the start of the academic period (UG1 and PG1). This finding is consistent with the findings of Kawabe et al. (2016), but contrary to the results of Gupta et al. (2018), who showed no significant relationship between IA and grade. A possible explanation for these two results is that as age increases during the college years, students may encounter increased cognitive maturity, which might explain the decline in IA. Contrastingly, it also gives more freedom to the students to make decisions regarding their behaviors (including Internet use), which might explain the increase in IA with increasing grades (Reta et al., 2020).

Two major limitations of the current study should be acknowledged. First, considering a large number of surveys in questionnaire distribution and collection, this study adopted the IADQ (created by Young in 1998 with only eight questions) to measure the level of IA of participants, which means that future studies are encouraged to utilize the newest questionnaires, like the Internet Addiction Test. Second, the distribution of academic status of the students in this study was unequal (most of them were undergraduate students); therefore, further studies may wish to expand the number of postgraduate students.

## Conclusion

Chinese college students with IA have a higher level of SA than students without IA, while students with a higher degree of SA have a greater likelihood of IA. Meanwhile, college students with more PE would have a lower level of SA and a lower probability of IA. The SA of single children was lower than that of non-single children. The SA level of female students was higher than that of men, while male students were more prone to IA. Students majoring in arts and sports have lower levels of SA than students majoring in other subjects, but students majoring in sports are more prone to IA. Students in the middle phase of their academic careers were more likely to have IA than those in the starting phase. This study helps to better understand the relationship between PE, SA, and IA in Chinese college students, which could offer a precise exercise prescription for those who suffer from SA and IA in different situations.

## Data Availability Statement

The raw data supporting the conclusions of this article will be made available by the authors, without undue reservation.

## Author Contributions

SY and HL contributed to the conception, design, and the examination of data of this research. HC and SY contributed to the collection and examination of data. SY and ZZ were responsible for writing. All authors contributed to the article and approved the submitted version.

## Conflict of Interest

The authors declare that the research was conducted in the absence of any commercial or financial relationships that could be construed as a potential conflict of interest.

## Publisher’s Note

All claims expressed in this article are solely those of the authors and do not necessarily represent those of their affiliated organizations, or those of the publisher, the editors and the reviewers. Any product that may be evaluated in this article, or claim that may be made by its manufacturer, is not guaranteed or endorsed by the publisher.
